# Exercise-induced pneumomediastinum

**DOI:** 10.1186/s12245-015-0089-9

**Published:** 2015-12-01

**Authors:** Tomoyuki Tobushi, Kazuya Hosokawa, Keita Matsumoto, Toshiaki Kadokami

**Affiliations:** Department of Cardiovascular Medicine, Saiseikai Futsukaichi Hospital, 3-13-1 Yumachi, Chikushino-shi, 818-8516 Fukuoka, Japan; Department of Cardiovascular Medicine, Kyushu University Hospital, 3-1-1 Maidashi, Higashi-ku, Fukuoka-shi, 812-8582 Fukuoka, Japan; Department of Respirology, Saiseikai Futsukaichi Hospital, 3-13-1 Yumachi, Chikushino-shi, Fukuoka, Japan

**Keywords:** Spontaneous pneumomediastinum, Chest pain, Emergency medicine, Sports medicine

## Abstract

**Background:**

A sudden onset of chest pain, which often reflects a life-threatening disease, requires prompt diagnosis in the emergency department.

**Findings:**

A 12-year-old boy presented with sustained chest pain and dyspnea after diving into a swimming pool and was transferred to our emergency department. A chest examination noted a crunching and rasping sound at the precordium, synchronous with the heartbeat. Chest radiography showed lucent streaks and the mediastinal pleura at the left cardiac outline. Additionally, computed tomography showed massive pneumomediastinum surrounding the heart. Thus, he was diagnosed with spontaneous pneumomediastinum.

**Conclusions:**

Spontaneous pneumomediastinum should be considered in the differential diagnosis of chest pain. In addition to medical history-taking, careful physical examination, which can identify the characteristic finding of a friction sound synchronous with the heartbeat (Hamman’s sound), will help in the immediate diagnosis of spontaneous pneumomediastinum.

## Findings

### Case synopsis

**Fig. 1:**
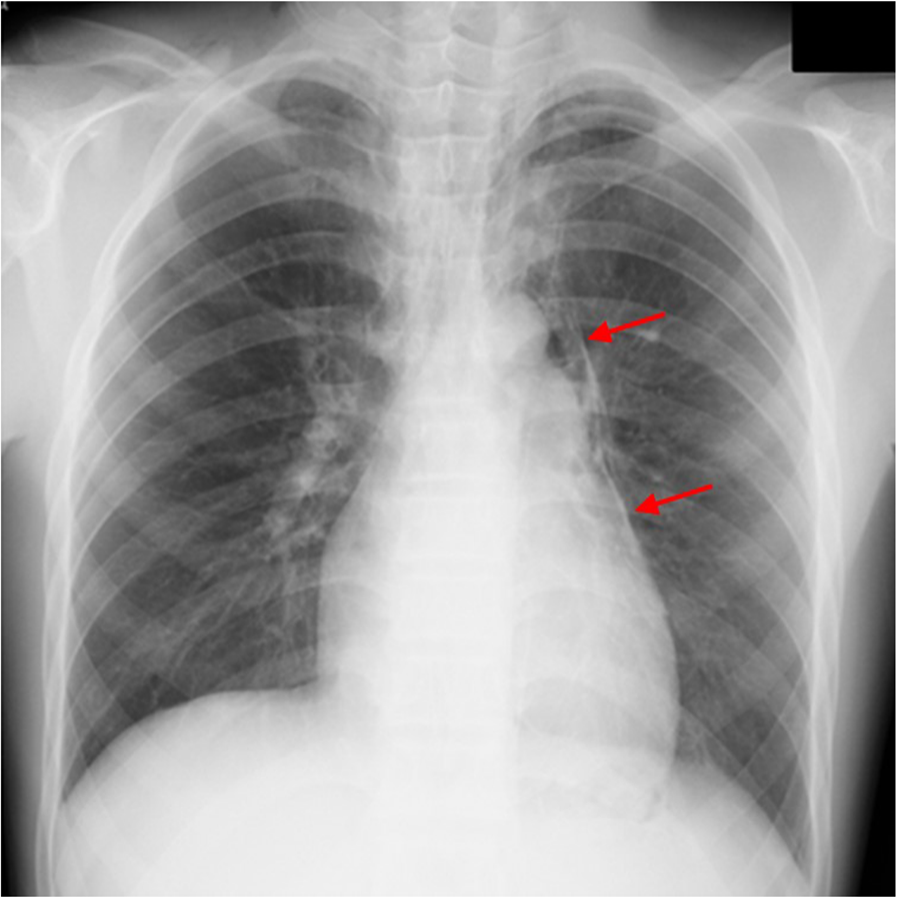
A chest radiograph of the mediastinal pleura

**Fig. 2:**
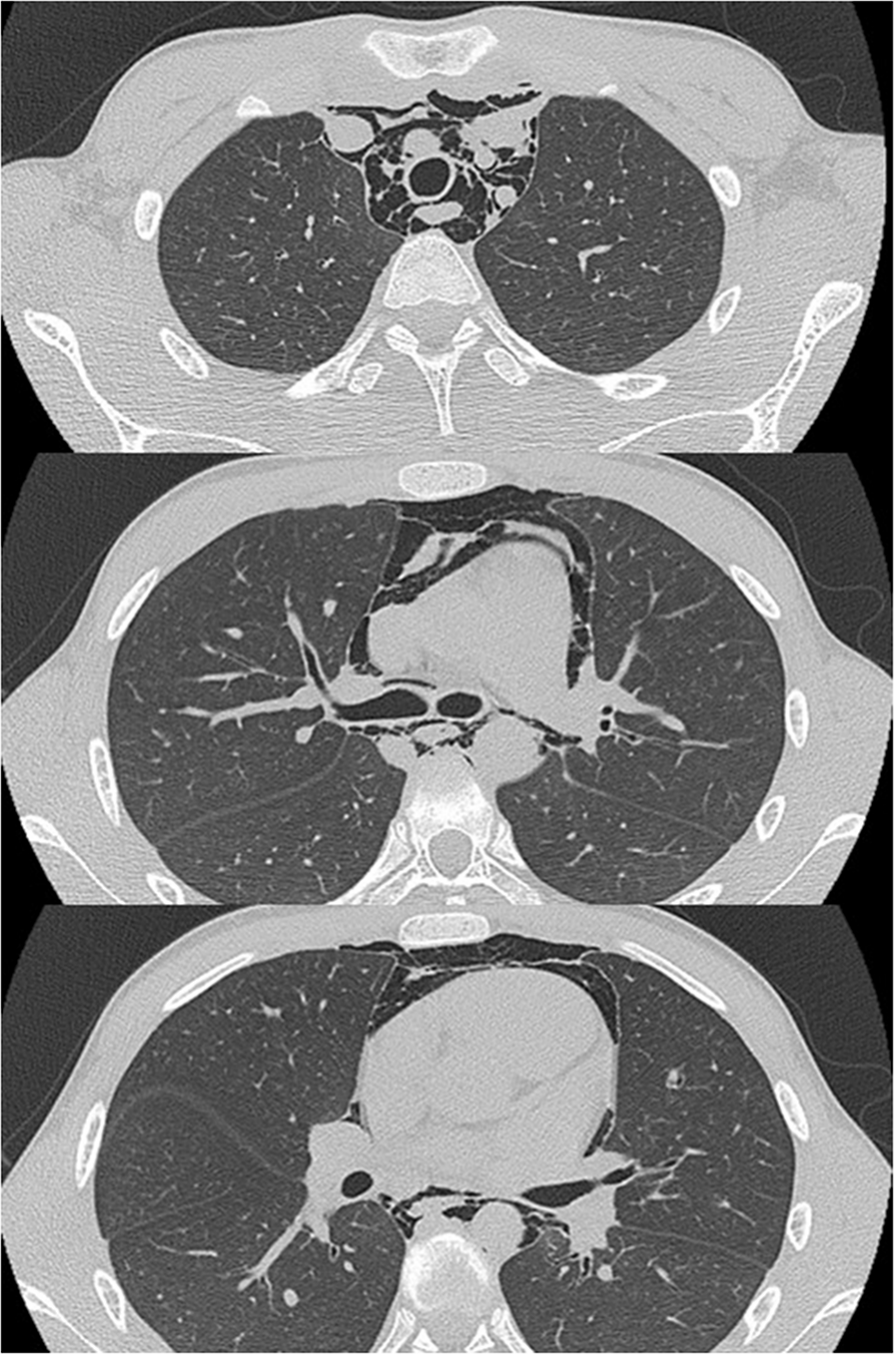
A computed tomography image showing massive pneumomediastinum surrounding the heart

A 12-year-old boy presenting with sustained precordial discomfort and dyspnea was transferred to our emergency department. Medical history-taking revealed that he suddenly experienced this discomfort immediately after diving into a swimming pool. His vital signs, including respiratory rate, blood pressure, heart rate, oxygen saturation, and body temperature, were within normal limits. A chest examination noted a crunching and rasping sound at the precordium, synchronous with the heartbeat. Electrocardiography showed no significant abnormalities, and blood analysis only showed a slight elevation of the white blood cell count. Chest radiography showed lucent streaks and the mediastinal pleura at the left cardiac outline (Fig. 1). Additionally, computed tomography showed massive pneumomediastinum surrounding the heart (Fig. 2). Thus, he was diagnosed with spontaneous pneumomediastinum (SPM). Esophagography ruled out esophageal perforation. The pneumomediastinum spontaneously reduced after 1 week of rest, and he completely recovered without any complications.

### Spontaneous pneumomediastinum

SPM is an uncommon condition in patients with chest pain (0.3 %) [1]. Most patients are male adolescents [2], and preexisting bronchial asthma primarily causes SPM. However, an exertional increase in airway pressure induced by daily activities (weightlifting or sports) often causes alveolar rupture, leading to SPM, even in the absence of underlying lung disease [3, 4]. Although most patients require hospitalization for observation or treatment (92 %), almost all are discharged within a few days, without further complications [5, 6]. However, as effort rupture of the esophagus (Boerhaave’s syndrome) or blunt chest trauma, which can cause pneumomediastinum, often requires intensive care [7, 8], it should be ruled out in the early period. SPM should be considered in the differential diagnosis of sudden chest pain, especially in young patients. In addition to medical history-taking, careful physical examination, which can identify the characteristic finding of a friction sound synchronous with the heartbeat (Hamman’s sound), will help in the immediate diagnosis of SPM.

## Abbreviations

SPM: spontaneous pneumomediastinum
